# Primary Mediastinal Dysgerminoma: A Case Report and Literature Review

**DOI:** 10.7759/cureus.57504

**Published:** 2024-04-03

**Authors:** Meryem El Jarroudi, Fatima Rezzoug, Soufia El Ouardani, Ouissam Al Jarroudi, Sami Aziz Brahmi, Said Afqir

**Affiliations:** 1 Medical Oncology, University Hospital Center Mohammed VI, Oujda, MAR; 2 Faculty of Medicine And Pharmacy, Mohammed First University, Oujda, MAR

**Keywords:** prognostic risk factors, mediastinum, chemotherapy, radiotherapy (rt), dysgerminoma

## Abstract

Germ cell tumors are malignant tumors that mostly develop in the gonads. Extragonadal localization is rare and may affect the mediastinal and sacrococcygeal regions. Mediastinal seminoma is a malignant germ cell tumor of the mediastinum. The tumor typically occurs in the anterosuperior mediastinum in males and often has a very slow growth pattern and limited potential for metastasis. And symptoms are not very characteristic, with many patients being asymptomatic and the tumor being discovered incidentally. In this paper, we report the case of a 26-year-old patient admitted for the management of a large anterosuperior mediastinal tumor encasing the vital structures of the mediastinum.

## Introduction

Germ cell tumors primarily originate from the gonads. However, extragonadal localizations such as the mediastinum, retroperitoneum, pineal gland, or coccyx are rare [1,2]. Primary mediastinal germ cell tumors represent 15% of anterior mediastinal cancers in adults [3], accounting for 1-6% of all mediastinal tumors [4]. The average age at diagnosis is 25 to 35 years [2]. Mediastinal germ cell tumors are divided into two main types: seminomas and non-seminomas, with non-seminomas being more prevalent. Seminomas have a better prognosis, with a 5-year survival rate of up to 90%, and are sensitive to chemotherapy and radiotherapy, unlike non-seminomatous tumors, which have a poorer prognosis and lower sensitivity to chemotherapy [4]. In this paper, we report the case of a 26-year-old patient admitted for the management of a large anterosuperior mediastinal tumor encasing the vital structures of the mediastinum.

## Case presentation

**Figure 1:**
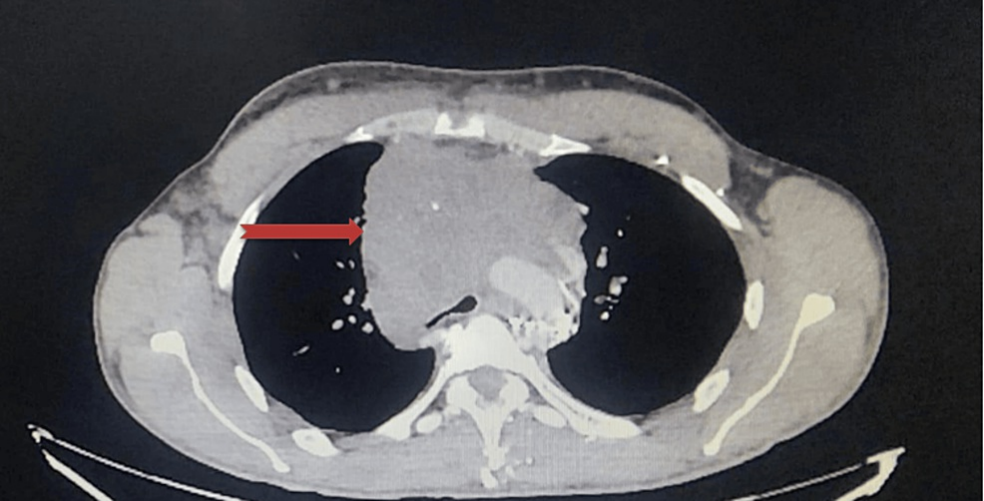
Thoracic CT scan showing large anterosuperior mediastinal tumor enveloping vital mediastinal structures (red arrow)

We report the case of a 26-year-old patient, with a history of multiple partners, admitted for management of stage II dyspnea with dry cough. Clinical examination revealed a conscious patient with a performance score (PS) of one. Cervical examination showed neck edema with collateral venous circulation and absence of palpable cervical lymph nodes. A computed tomography (CT) scan revealed a large anterosuperior mediastinal tumor encasing the vital structures of the mediastinum causing total thrombosis of the superior vena cava and cervical and axillary lymphadenopathy (Figure 1).

**Figure 2:**
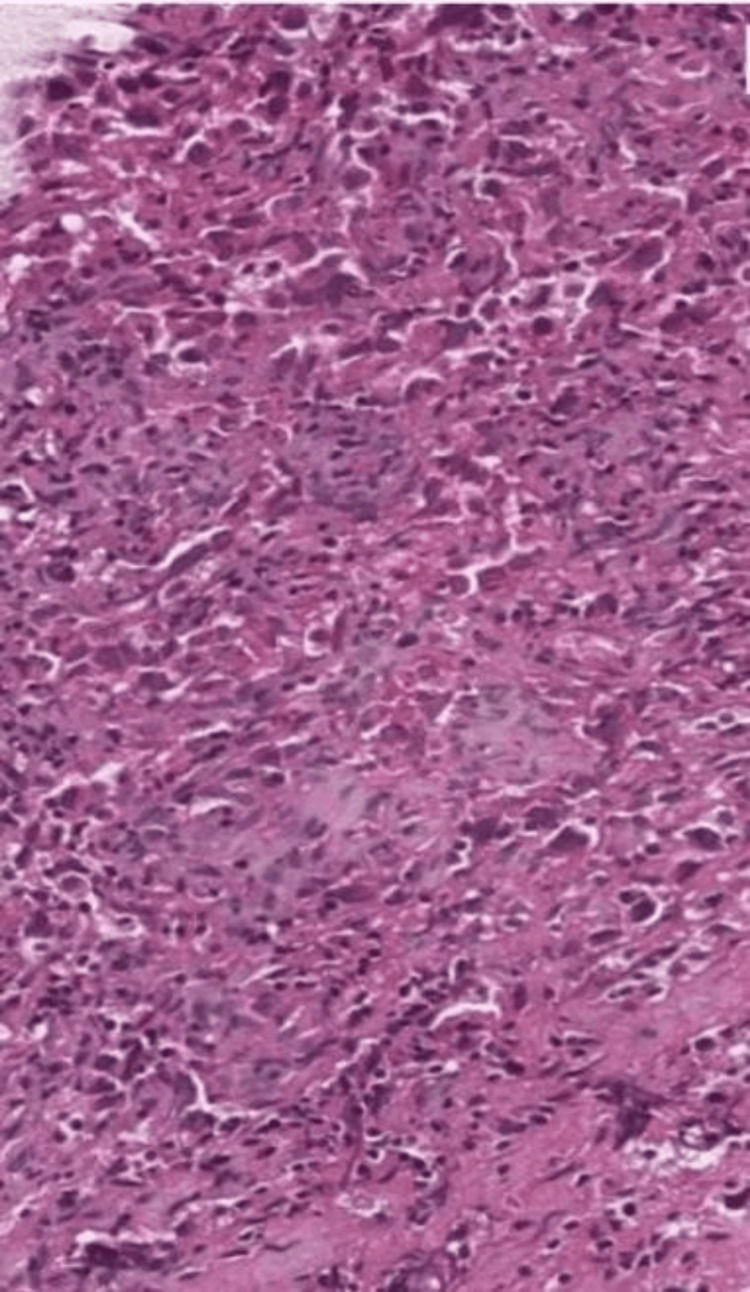
Round cell proliferation with fibrous stroma Staining: Hematoxylin-eosin Magnification: 20x

**Figure 3:**
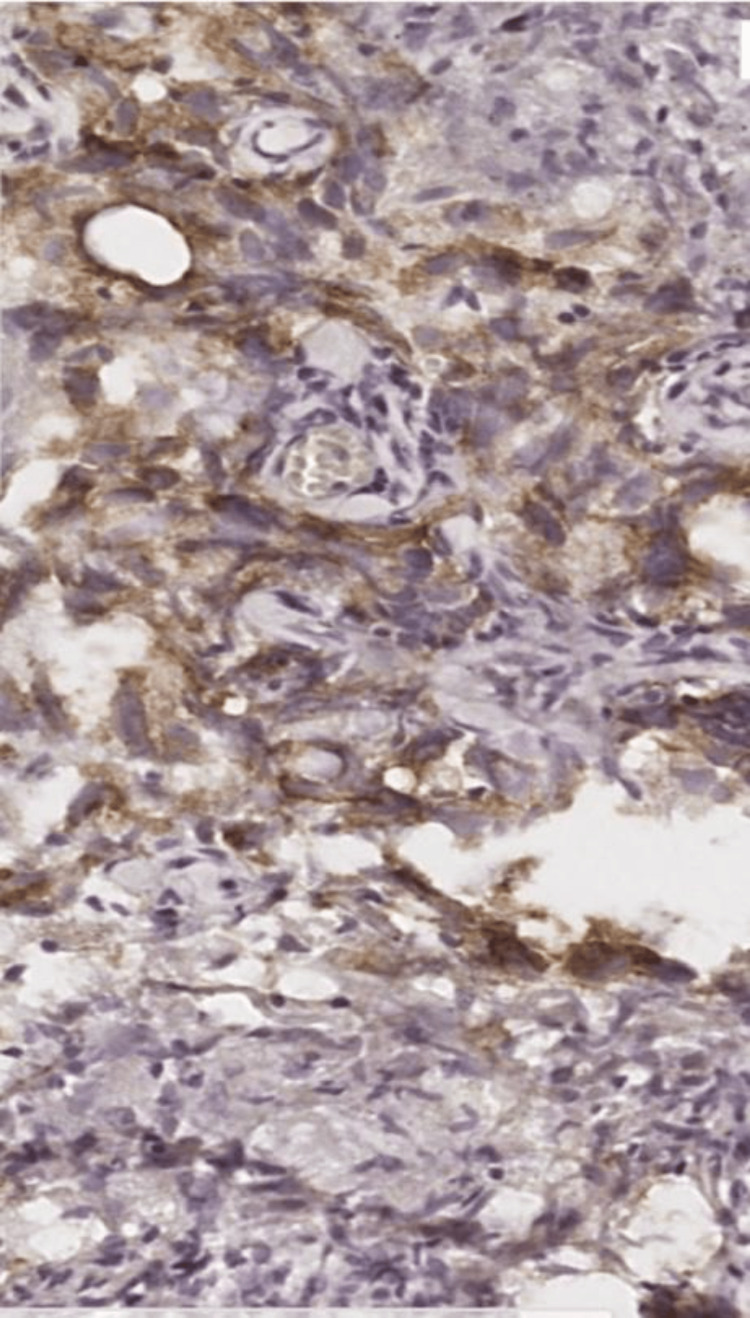
Immunohistochemical study showing positivity of anti-placental alkaline phosphatase (PLAP) antibody

**Figure 4:**
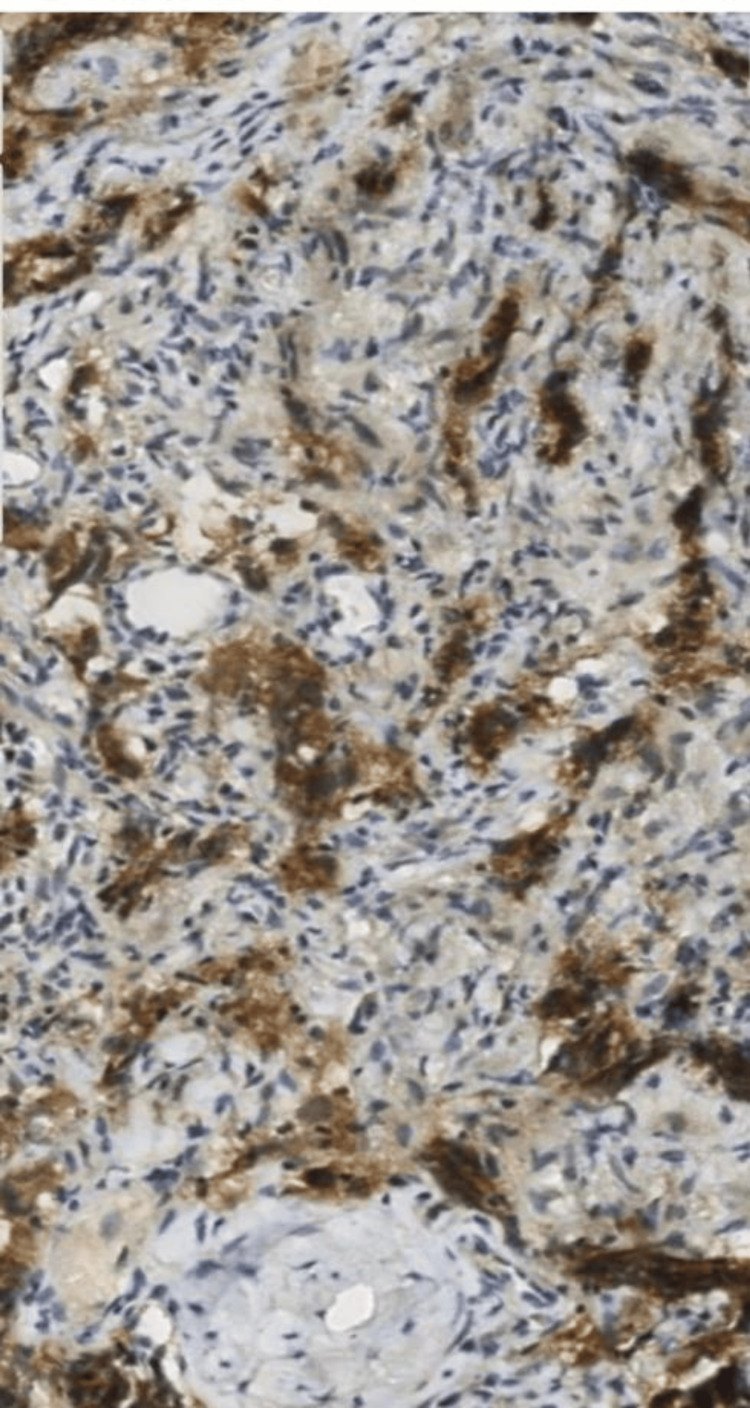
Immunohistochemical study showing positivity of CD11

A transthoracic biopsy showed a malignant round cell tumor, immunohistochemistry confirmed a mediastinal dysgerminoma (cytokeratin CK-, CK7, CK5/6-, cluster of differentiation CD30, CD15, CD20, CD3-, anaplastic lymphoma kinase ALK-, CKIT+) (Figures 2-4) with negative serum tumor markers in particular beta-human chorionic gonadotropin (bHCG), alpha-fetoprotein (AFP), and lactate dehydrogenase (LDH).

The case was discussed at our multidisciplinary tumor (MDT) board meeting, where the consensus was for the patient to four cycles of BEP chemotherapy: bleomycin 30 units on days 1, 8, and 15, etoposide 100 mg/m^2^ daily on days 1-5, cisplatin 20 mg/m^2^ daily on days 1-5. After the second cycle, the patient presented with superior vena cava syndrome and underwent radiotherapy at the mediastinal level with a dose of 40 Gy in 20 fractions of 2 Gy, over 33 days, resulting in clinical improvement. After completing chemotherapy, a follow-up CT scan showed a decrease in the size of the anterosuperior mediastinal tumor, now measuring 76x65x36 mm compared to its initial size of 102x95x86 mm. The tumor was in contact with the vascular axes without invading them or causing compression. The superior vena cava and the azygos vein remained patent and of normal caliber. These findings suggest a partial tumor response. A positron emission tomography (PET) scan showed a non-hypermetabolic, ill-defined, anterosuperior mediastinal mass, deemed unsuitable for surgical intervention due to its size. The patient was placed under surveillance. Two years of post-therapy surveillance, including CT and clinical examinations every three months, along with tumor marker tests, have been unremarkable. Our patient is still alive at the time of writing this case report with a good performance status PS=1. Additionally, the patient was satisfied with our management.

## Discussion

Germ cell tumors typically originate from the gonads, being most commonly observed in the testicles in males and more rarely in the ovaries in females. Extragonadal localization is rare and may include areas such as the mediastinum, retroperitoneum, pineal region, and suprasellar region [2,5]. Primary mediastinal germ cell tumors are a rare entity, representing 15% of all tumors localized in this area, accounting for 1-6% of all mediastinal tumors [3,4], with an average age at diagnosis ranging from 25 to 35 years [3,6].

Germ cell tumors are divided into two subgroups, namely seminomatous and non-seminomatous tumors. Non-seminomatous tumors include choriocarcinomas, embryonal carcinomas, yolk sac tumors, teratomas, and mixed germ cell tumors [7,8].

Mediastinal germ cell tumors are generally symptomatic, with clinical symptoms including cough, chest pain, dyspnea, chills, fever, and superior vena cava syndrome [9,10]. In our case, the reported clinical symptoms were dyspnea and cough, in imaging studies, mediastinal seminomas have been described as large, voluminous, well-defined, and lobulated, often extending on either side of the midline. Mediastinal seminomas also exhibit areas of low attenuation and ring-like and punctate calcifications [11].

Additionally, Moran and Suster analyzed 322 patients, with the majority of cases occurring in males. The results of this study showed that mediastinal germ cell tumors have demographic and histopathologic distributions similar to those of tumors occurring in the male gonads, with teratomatous and seminomatous lesions being the most common [7].

Histologically, seminomatous germ cell tumors present with cells with a distinct cell membrane and abundant clear eosinophilic cytoplasm containing glycogen [12]. On immunohistochemistry, seminomas tested positive for placental alkaline phosphatase (PLAP), octamer transcription factor-4 (OCT-4), c-tyrosine-protein kinase (c-Kit), and sal-like protein-4 (SALL-4) and negative for CD30 and cytokeratin [13].

Oncology societies recommend treating primary germ cell tumors similarly to gonadal germ cell tumors according to the classification of the International Germ Cell Cancer Collaborative Group (IGCCCG) [2]. Patients with seminoma are often treated with a regimen comprising three to four cycles of BEP regimen (bleomycin, etoposide, and cisplatin (BEP)) depending on the IGCCCG risk group.

Seminomatous germ cell tumors are classified according to the IGCCCG into two groups characterized by a normal AFP regardless of hCG and LDH: the first one is associated with a good prognosis and absence of extrapulmonary visceral metastases. The second group is of intermediate risk with the presence of extrapulmonary metastases.

Our patient is classified according to the TNM classification: stage III, and according to the IGCCCG score has a good prognosis. The European Society for Medical Oncology (ESMO) recommends treating stage III diseases with treatment consisting of 3 to 4 cycles of BEP [14].

Mediastinal seminomatous germ cell tumors are tumors with a higher probability of response to chemotherapy and a better prognosis [15]. Prognostic factors reported based on a retrospective analysis of 31 patients included histological type, surgical resection of the tumor, and less than 10% viable tumor cells present in the resection material, as well as a classification according to the IGCCCG as having a good prognosis [16]. This classification allows risk stratification as good or intermediate based on non-pulmonary visceral metastases. Seminomas have excellent cure rates regardless of mediastinal or testicular localization, with an overall 5-year survival rate exceeding 90%. Extrapulmonary metastases or metastases at two or more sites are poor prognostic factors [1]. Tumor markers are not used to attribute prognosis in seminomas [14].

An update from the IGCCCG suggests that LDH levels exceeding 1.5 times the normal range are linked to a poor prognosis in patients with mediastinal seminomatous germ cell tumors [17].

## Conclusions

Primary mediastinal seminomas are rare tumors that are morphologically similar to their testicular counterparts but may exhibit different biological behavior due to their unique anatomical location. Prompt diagnosis and treatment are crucial in managing germ cell tumors, and multimodal therapy, including chemotherapy, is often necessary to control the tumor. Due to the high risk of metastasis, vigilant monitoring for recurrence is essential.
